# Every Vessel Counts: Neural Network Based Maritime Traffic Counting System

**DOI:** 10.3390/s23156777

**Published:** 2023-07-28

**Authors:** Miro Petković, Igor Vujović, Nediljko Kaštelan, Joško Šoda

**Affiliations:** Faculty of Maritime Studies, University of Split, Ruđera Boškovića 37, 21000 Split, Croatia; ivujovic@pfst.hr (I.V.); nkastelan@pfst.hr (N.K.); jsoda@pfst.hr (J.Š.)

**Keywords:** maritime traffic counting, video surveillance, YOLOv4, Kalman tracker, non-AIS vessels

## Abstract

Monitoring and counting maritime traffic is important for efficient port operations and comprehensive maritime research. However, conventional systems such as the Automatic Identification System (AIS) and Vessel Traffic Services (VTS) often do not provide comprehensive data, especially for the diverse maritime traffic in Mediterranean ports. The paper proposes a real-time vessel counting system using land-based cameras is proposed for maritime traffic monitoring in ports, such as the Port of Split, Croatia. The system consists of a YOLOv4 Convolutional Neural Network (NN), trained and validated on the new SPSCD dataset, that classifies the vessels into 12 categories. Further, the Kalman tracker with Hungarian Assignment (HA) algorithm is used as a multi-target tracker. A stability assessment is proposed to complement the tracking algorithm to reduce false positives by unwanted objects (non-vessels). The evaluation results show that the system has an average counting accuracy of 97.76% and an average processing speed of 31.78 frames per second, highlighting its speed, robustness, and effectiveness. In addition, the proposed system captured 386% more maritime traffic data than conventional AIS systems, highlighting its immense potential for supporting comprehensive maritime research.

## 1. Introduction

Video surveillance systems in ports play an important role in various aspects of maritime operations, from safety and security to efficient port management. Furthermore, the accurate tracking and counting of vessels provide researchers with data to analyze traffic density and patterns, assess environmental impacts, optimize vessel routes, and more. Today, traditional automatic maritime vessel counting systems rely on systems, such as the Automatic Identification System (AIS) and Vessel Traffic Services (VTS), since manual counting is labor-intensive and time-consuming. However, relying solely on AIS and VTS to monitor vessels has limitations. Not all vessels are required to have AIS on board, resulting in incomplete data [1], and it can be difficult for VTS to accurately identify and track vessels in crowded or complex port environments. This problem is particularly relevant in Mediterranean passenger ports such as the Port of Split, where maritime traffic is diverse and consists of a large number of vessels that are not covered by traditional vessel monitoring systems [2]. To overcome these limitations, advances in computer vision combined with video surveillance have emerged as a promising solution. They offer efficiency, accuracy, and reliability in obtaining detailed information about maritime traffic in a particular maritime zone in real time [3]. The availability of accurate and automated vessel counting systems from land-based cameras opens up opportunities for more extensive research and applications in the maritime domain.

### 1.1. Related Work

The ARGOS project is one of the pioneering projects for video-based maritime traffic monitoring systems [4]. This project installed several static, land-based cameras along the Grand Canal in Venice in 2007, which enabled the detection and tracking of vessels. The system had vessel counting capabilities but had problems tracking fast-moving objects and could not classify vessels. Later enhancements of the same system introduced a non-neural network classifier for ship classification [5]. While the system achieved over 80% counting accuracy, it was not evaluated on the real number of vessels passing through the monitored zone. In [6], an NN based object detector is used for vessel target tracking and self-correction counting in waterway scenes using YOLOv3. To overcome the challenges of target drift and jitter, the authors propose a self-correcting network that combines regression-based direction determination and a target counting method with variable time windows. The proposed system shows stable counting performance despite complex scenarios and weather conditions. However, it is important to note that the system is trained to detect a single general class for all vessels. Recently, in [7], YOLOv5 was used as an object detector and a simple Kalman tracking algorithm to detect and count ships via a webcam in Manilla Bay, Philippines. The system was trained for five classes of vessels (Bulk Cargo Carrier, Container Ship, Fishing Boat, General Cargo Ship, and Passenger Ship). Some of the misclassifications are due to the high similarity of the different classes and the underrepresentation of some classes. It can be observed that the produced model is accurate in detecting, classifying, and counting ships based on their type.

Based on the analysis of existing studies, the number of papers in the field of maritime vessel counting is limited. However, the existing studies emphasize the importance of vessel detection and tracking as essential components of the counting process.

Advances in neural network (NN) based object detectors have led to numerous papers combining them with various sensors to monitor maritime traffic. Satellite imagery is a popular tool for monitoring large areas [8], where multiple NNs of the YOLO family are trained and compared. The authors used them to detect maritime vessels and land vehicles in land and sea images. The average accuracy achieved for maritime vessels was 60%. In [9], Side-Looking Airborne Radar (SLAR) images were used as input to a two-stage CNN to detect ships and oil spills. In [10], a multi-stage data-centric workflow integrating optical satellite imagery and AIS data for automatic vessel detection is presented. This hybrid approach resulted in real world accuracy of 86–90%. In [11], UAV was used to monitor the maritime environment. The authors tested different architectures for their efficiency, accuracy, and speed.

A review in [12] focuses on summarizing the methods and application scenarios of maritime object detection, analyzes the characteristics of different maritime datasets, highlights the marine detection application of the YOLO series model, and also discusses the current limitations of object detection based on Deep Learning (DL) and possible breakthrough directions. In [13], the authors used freely available aerial image dataset to train and test the two popular single-stage object detection models, YOLOv4 and Tiny YOLOv4. The trained models were evaluated on different ship images, and detections were performed. As a result of the study, Mean Average Precision (mAP) values of 80.82% and 62.30% were obtained with the YOLOv4 and Tiny YOLOv4 architectures, respectively. The lack of fast ship detectors that detect multiple ship classes is addressed in [14] with a real-time ship detector based on Fast U-Net and remapping Attention (FRSD) via a common camera. In [15], LSDM was used to improve the accuracy of YoloV3. The goal was to improve the accuracy and speed of ship detection.

In [16], extensive research was conducted on approaches for tracking objects in maritime environment. The authors pointed out the advantages of the Kalman filter (KF) for tracking objects at sea, as this algorithm works in real time and can handle complex, slow-moving variations but requires a dedicated framework for tracking multiple objects. This tracking algorithm can tolerate errors in detection by using the prediction step of KF to improve performance in a video sequence [17]. The effectiveness of KF is evaluated in [18] when it is used as a method for tracking multiple objects in cluttered situations. Moreover, in [4], a KF-based multi-hypothesis tracker was implemented for video surveillance, which has multi-target capabilities and provides a good balance between real-time requirements and robustness. In [4,18], it is shown that KF tracking is an optimal solution that is easy to implement and computationally undemanding. A KF and Hungarian assignment (HA) algorithm is proposed for fast tracking of multiple objects [19]. This approach proved to be very robust and not computationally demanding. The authors in [20] evaluated several convolutional NNs as detectors with KF and Hungarian assignment as tracker and concluded that the tracking performance of KF depends on how accurately the detector localizes the targets. A similar approach was used in [21] and verified on a dataset focusing on occlusion conditions, proving that it can handle occlusion problems.

The Section 1 highlights the significance of NN object detection and tracking in maritime environments. Notably, limited papers focus on maritime vessel counting, but they emphasize the importance of vessel detection and tracking as essential components of the counting process. Studies have explored the capabilities of convolutional NNs and YOLO models for marine detection, pointing out challenges like complex environments, scale variations, cluttered backgrounds, and low-resolution images within datasets. Tracking algorithms such as KF and HA have shown effectiveness in multi-object tracking while the accuracy of tracking relies on the detector’s localization performance.

### 1.2. Problem Statement

In this paper, we focus on the Mediterranean maritime zone, in particular the port of Split. This maritime zone presents unique challenges for accurate traffic monitoring and vessel counting due to the specific configuration of maritime traffic. As described in [2], due to the port structure, traffic ranges from smaller boats of all types, such as sailboats and speedboats to passenger ferries and large cruise ships. Traditional traffic monitoring systems have proven inadequate for monitoring and counting this variety of vessels, resulting in an incomplete or inaccurate traffic analysis [1]. The ability to accurately count vessels provide valuable data for scientific research, such as maritime traffic density analysis [22], collision probability modeling [23], or environmental monitoring [24].

**Figure 1:**
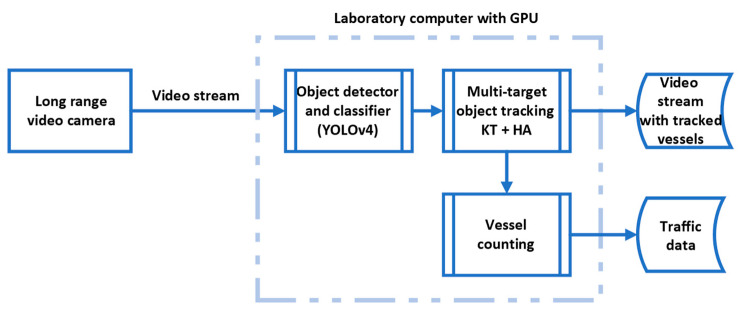
Illustration of the proposed system.

The main objective of this paper is to propose a methodology, shown in Figure 1, that meets the specific requirements of these maritime zones. The proposed system provides accurate and automated vessel counting from static land-based cameras by using YOLOv4 as an object detector (Section 2.2) and Kalman filter with Hungarian association as a multi-target tracker (Section 2.3).

The main scientific contributions can be summarized as follows:

- Enhanced counting and classification of vessels into 12 categories as they traverse the maritime zone, utilizing an adjustable counting zone that differentiates between vessels entering and leaving the port;
- Introduction of stability assessment as a complement to tracking algorithm to eliminate of false positives on unwanted objects, such as seagulls flying in front of camera, reflections from sea surface, etc.;
- Training and validation of YOLOv4 on the newly compiled SPSCD [2] dataset.

This paper is organized as follows: the Section 2 presents methodology and settings, and the Section 3 present results. Finally, a discussion and conclusions are given.

## 2. Methodology and Settings

This section presents the methodology and settings used for developing a vessel counting system. The first subsection provides information about the long-range video camera, its location, and computer specifications. The second subsection covers the object detector training while the third subsection provides the information about the multi-target tracking algorithm.

### 2.1. Hardware Settings

A Dahua DH-TPC-PT8620A-T [25] surveillance camera was acquired as a project component and installed on the building overlooking the port’s entrance to monitor all incoming and outgoing maritime traffic at the Port of Split. The camera was positioned at the following coordinates: Latitude 43°30′04″ N, Longitude 16°25′48″ E, and was mounted at a height of 9 m above sea level. Then, the video stream with H.264 compression, 1920 × 1080 resolution, and 25 frames per second (FPS) was routed to a laboratory computer. The computer specifications are provided in Table 1.

### 2.2. Object Detector

To detect maritime targets in real time, the convolutional neural network You Only Look Once Version 4 (YOLOv4) [26], written in C, was used as an object detector and classifier. For model training, publicly available pre-trained weights were obtained. Then, the model was trained on the Split Port Ship Classification Dataset (SPSCD) [2]. The dataset consisted of 19,337 images in 1920 × 1080 resolution with 27,849 labeled ships, categorized into 12 classes. For reference, examples of each class can be found in Appendix A. The dataset contained images taken under different weather conditions (e.g., rainy, cloudy, sunny, foggy, …), different illumination effects from the sun and sea surface reflections (different times of the year and day), and different sea state conditions [2]. In addition, the vast majority of the images contained the port and starboard sides of the ships. Then, 2339 images (or 12% of the total) were selected for the validation set while the rest were used for the training set. The input size of the image was set to 608 × 608 pixels with a batch size of 64 while the maximum number of batches was set to 24,000. Moreover, the learning rate was set to 0.001, decay to 0.0005, and momentum to 0.949, and the mosaic data augmentation technique was used during the training. Then, model was evaluated on validation set with metrics described in [27], and achieved results are presented in Table 2.

**Figure 2:**
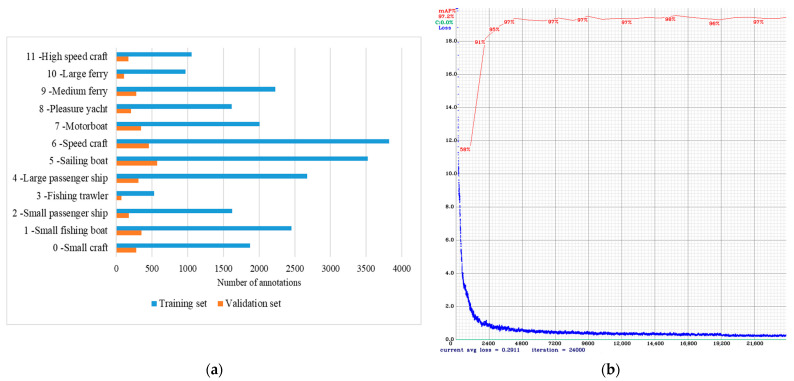
(**a**) Distribution of class annotations in the training and validation set; (**b**) Loss and accuracy graph of the YOLOv4 model trained on the SPSCD dataset.

The detailed distribution of class annotations in the training and validation set is presented in Figure 2a, where the horizontal axis shows the number of annotations and the vertical axis shows the class identification number and class name.

**Figure 3:**
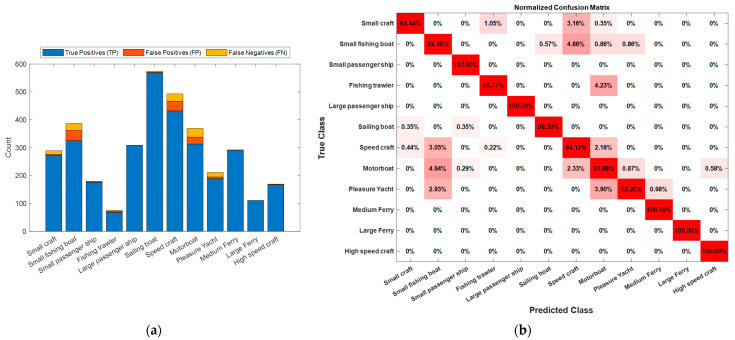
(**a**) Distribution of class detections; (**b**) Normalized confusion matrix.

The mAP value and the average loss of the model, achieved during the training, is presented in Figure 2b, where the horizontal axis of the graph indicates a number of iterations while the vertical axis indicates the loss value. The blue line represents the loss value, and the red line indicates the mAP value. In order to compare the models performance across multiple classes, we utilized a normalized confusion matrix presented in Figure 3b, which focused on the proportion of correct and incorrect predictions within each class. In addition, distribution of True Positives (TP), False Positives (FP), and False Negatives (FN) detections are presented in Figure 3a.

The diagonal entries on Figure 3b, represent the proportion of instances that were correctly classified. For example, when observing the true class, ‘Small fishing boat’ was represented in the second row and column of the matrix. The diagonal entry of 92.86% indicated the proportion of TP, i.e., ‘Small fishing boat’, instances correctly identified as such. Within the same row, the entries 0.57%, 4.86%, 0.86%, and 0.86% represented instances of ‘Small fishing boat’ incorrectly predicted as ‘Sailing boat’, ‘Speed craft’, ‘Motorboat’, and ‘Pleasure yacht’, respectively. Finally, examining the second column provided insights into instances incorrectly identified as ‘Small fishing boat’. The values 3.05%, 4.94%, and 2.93% in this column corresponded to ‘Speed craft’, ‘Motorboat’, and ‘Pleasure yacht’ classes, respectively.

### 2.3. Object Tracking

**Figure 4:**
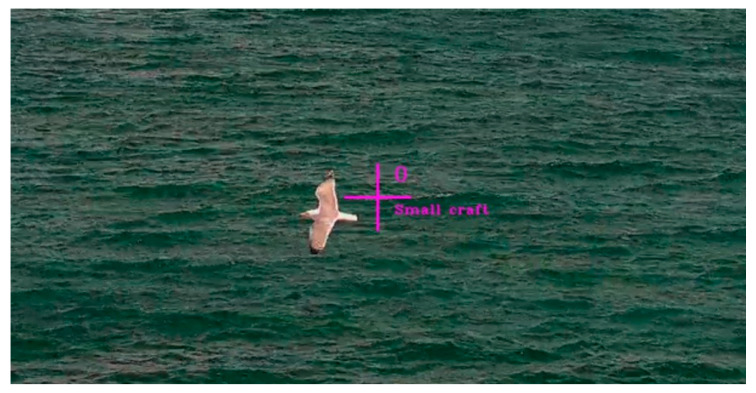
Example of a false positive detection.

Object tracking could be described as follows. When a maritime target was detected by an object detection system, the subsequent task of a tracking algorithm was to estimate the position and parameters of the moving target. In addition, the object detection system might not have consistently detected the target in every frame, so the tracking algorithm had to process intermittent detections. A multi-target tracker based on the Kalman Filter (KF) with the Hungarian Association (HA) was selected for its robustness and real-time performance to bridge gaps in detections. The KF was used for state estimation while the HA facilitated the association of detections to existing tracks. During the evaluation of the object detection and tracking system, we observed instances where YOLOv4 incorrectly identified seagulls and/or reflections on the sea surface as objects of interest. Although the confidence threshold was set relatively high, FP detections occurred and were consequently tracked by the tracking algorithm, as shown in Figure 4.

Multiple video sequences with FP tracks were examined, specifically looking at the time period between the first FP detection and the end of the tracking. Thus, it was calculated that they were usually detected in 5% of the total frames in this time frame. In addition, video sequences with TP tracks were examined under various weather, sea state, and illumination conditions. When looking at the time period between the first TP detection and the end of tracking, it was calculated that TP was typically detected in 30% to 90% of the total frames in this time period. The results of these observations were used to introduce an improvement that will complement the tracker to eliminate the FP tracks and increase the stability of the tracking process.

**Figure 5:**
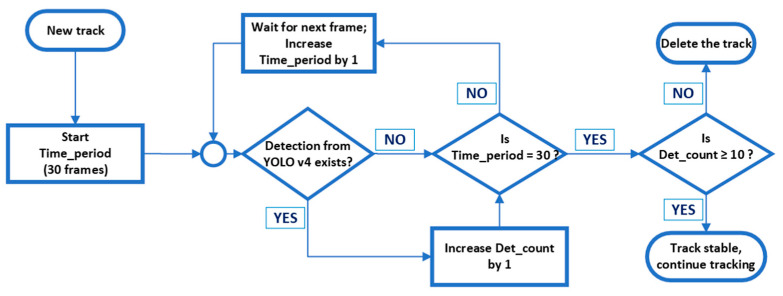
Flowchart of proposed tracking stability assessment.

This improvement involved evaluating the consistency of the detections based on a predefined criterion that must be met over a certain period of time (measured in frames), as shown in Figure 5.

The time period for the track stability assessment was empirically set to 30 frames. When YOLOv4 detected the new track for the first time, the number of subsequent detections was monitored. If the number of detections after 30 frames was equal to or exceeded predetermined threshold, the track was considered stable, the bounding box was generated, and tracking continued. On the other hand, if the number of detections after 30 frames was less than the threshold, the track was discarded. As a result of the observations mentioned in the previous paragraph, the threshold was set to 10 detections per time period (30% of frames). It should be noted that KF and HA were utilized from the beginning of the track while the proposed stability assessment was performed only when new tracks were detected.

A performance evaluation of object tracking algorithm, adopted from [28], was used for improvement validation. Motion tracking was defined as the problem of estimating the position and the spatial extent of the tracked objects in each frame of a video sequence. The result was a set of tracks T_j_, j = 1, …, M, for all M tracked objects of the scene while track T_j_ was defined as [28]:

(1) $${\;T}_{j}=\left\{x_{\mathrm{ij}}\;,{\;B}_{\mathrm{ij}}\right\}$$

where x_ij_, and B_ij_ were the center and the spatial extent (represented by a bounding box) of the object j in the frame i, and N was the number of frames.

Spatial and temporal overlap between tracks was used to quantify the matching level between the Ground truth Track (GT) and System Track (ST). The spatial overlap was defined as the overlapping level A(GT_i_, ST_j_) between GT_i_ and ST_j_ in a specific frame k [28]:

(2) $$A\left({\mathrm{GT}}_{\mathrm{ik}},{\mathrm{ST}}_{\mathrm{jk}}\right)=\frac{\mathrm{Area}\left({\mathrm{GT}}_{\mathrm{ik}}\cap {\mathrm{ST}}_{\mathrm{jk}}\right)}{\mathrm{Area}\left({\mathrm{GT}}_{\mathrm{ik}}\cup {\mathrm{ST}}_{\mathrm{jk}}\right)},$$

For validation, a video sequence with one True Positive (TP) and one False Positive (FP) was selected, and the GT was manually generated. Spatial and temporal overlap between the tracks, with and without the tracking stability assessment, is presented in the Results section.

### 2.4. Object Counting

**Figure 6:**
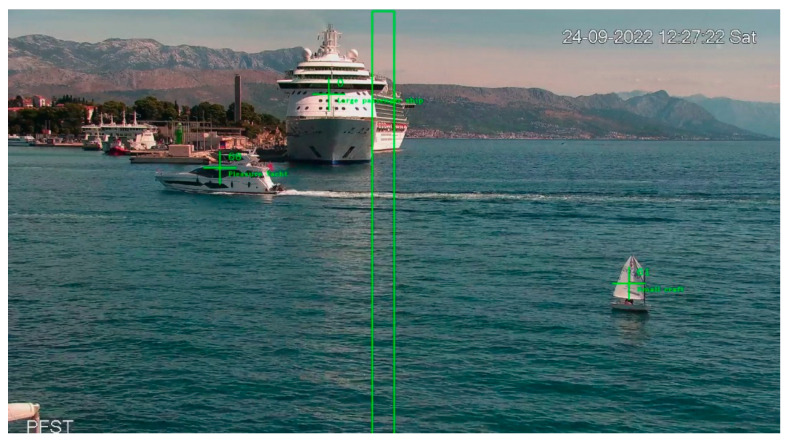
Camera FoV, with the green rectangle in the center indicating the defined counting zone.

Since not every vessel that appeared in the camera’s Field of View (FoV) intended to enter or leave the port of Split, a manually adjustable counting zone was added. Furthermore, the counting zone could be set in any shape as long as it maintained the shape of a polygon, as shown in Figure 6.

The purpose of the counting zone was to count tracked targets in such way that when a center of the bounding box enters the zone, we increased the number of vessels by one for that specific class. In addition, each individual track could only be counted once. To verify that the tracked target was within the counting zone, the following procedure was used:

1. From the center of the track, draw a horizontal line to the right, extending it to infinity;
2. Count how many times the horizontal line intersects with the edges of the counting zone;
3. If the number of intersections is odd, the target is inside the counting zone or at its edge. If the number of intersections is even or zero, the target is outside the counting zone.

It was also important to determine whether the counted vessel was entering or leaving the port. For each counted target, the relative velocity in the *x*-axis (from the KF state vector) was examined. If the sign of the relative velocity was positive, the tracked target was moving from the left side of Figure 5 to the right, thus leaving the port. If the sign was negative, the tracked target moved from the right side of Figure 6 to the left, i.e., it entered the port.

Since the main goal of the vessel counting system was to provide traffic statistics based on categories of boats [5], the performance metrics for the counting accuracy were computed as follows:

(3) $$\mathrm{counting}\;\mathrm{accuracy}=1-\frac{\left|\hat{n}-n\right|}{n},$$

where n was the GT while $\hat{n}$ was the estimated count provided by the vessel counting system.

## 3. Results

### 3.1. Tracking Algorithm Improvement

**Figure 7:**
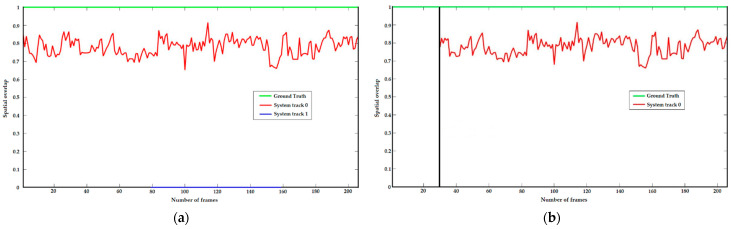
(**a**) Spatial and temporal overlap between tracks without stability assessment; (**b**) Spatial and temporal overlap between tracks with stability assessment.

To validate the proposed tracking stability assessment, a 210-frame video sequence is selected in which the vessel travels parallel to the camera and leaves the port. In addition, the video sequence contains several problems with the illumination and reflections of the sea surface. Figure 7 shows the spatial and temporal overlap between the tracks with and without tracking stability assessment.

Figure 7a shows the spatial overlap without stability assessment with the green line representing the GT track, the red line the TP, and the blue line representing the FP track. The FP track is detected at frame 79 due to reflections on the sea surface and is tracked by the tracking algorithm until frame 159. Moreover, Figure 7b shows the spatial overlap between the tracks with the stability assessment, where the green line represents the GT and the red line represents the TP track, while the black vertical line represents the end of the assessment period.

### 3.2. Vessel Counting

In order to validate the performance of the vessel counting system, a video sequence of maritime traffic during the peak summer season was recorded. This sequence spans a duration of 3 h and 10 min, specifically from 9:25 to 12:35, during which the port of Split experiences high and diverse traffic. Additionally, the recorded video contains changes in illumination, transitioning from partially cloudy to full sunlight. To gain a general picture of the maritime traffic volume for this time span, data were collected from AIS and manually counted, while the resulting counts are shown in Table 3.

Table 3 shows that, according to AIS, the total number of vessels entering the port is 14 and the total number of vessels leaving the port is 20 while the total number of vessels is 34. As can be seen from first column, AIS does provide information on vessel category, but these categories are too general or sometimes unknown. For example, when a vessel is identified as “Passenger (A)”, it means that it is known to be a passenger’s vessel, but the system does not provide further details about whether it is a cruise ship, ferry, or another specific type of passenger’s vessel. In addition, the designation “Unspecified (A)” indicates that the type of vessel is unknown or not provided in the transmitted AIS message with the letter “A” serving as a placeholder for the absence of a specific vessel type.

In the same video sequence, a human expert manually counted the vessels entering and leaving the port. This manually derived data serve as the GT for evaluating the performance of the vessel counting system. A comparative analysis between the manual counts and the best automated counts from our proposed system is provided in Table 4.

**Figure 8:**
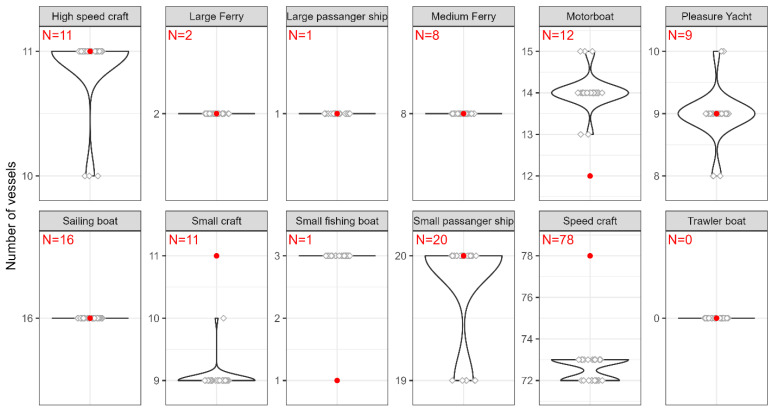
Distribution of vessel counts for each vessel category.

To simplify the evaluation for each vessel category, the number of vessels entering and leaving the port were summarized to obtain a total count. The counting process was then repeated 30 times to identify discrepancies between the results. The results are shown as a violin plot in Figure 8, where each box shows the vessel counts distribution for each vessel category.

In Figure 8, the *y*-axis represents the number of counted vessels while the number N and the red dot represent the GT. The width of each violin corresponds to the density of count data points (white dots), with wider sections indicating a higher concentration of counts. In plots where the violin is represented by a horizontal line instead of the usual shape, it can be seen that there is no variability in the count data for that particular category. For example, in the ‘Trawler boat’ category box, the horizontal line indicates that all count results consistently resulted in 0 vessels, which corresponds to the number 0 of GT. In the ‘Pleasure Yacht’ box, 26 of the 30 count results showed 9 vessels, resulting in a wider section of the violin plot for that count value. However, two count results displayed 8 and 10 vessels, respectively, resulting in a narrower section of the violin plot for these specific counts.

Statistical measures are calculated, including the minimum, maximum, average, median, and standard deviation (STD). In addition, the counting accuracy for each vessel category is determined using the average values. The results are presented in Table 5.

An additional statistical analysis was performed, and it was found (using the Kolmogorov-Smirnov test) that the data distribution was not normal. This is explained by the fact that ships, such as ferries (with AIS on-board), enter the port according to a specific schedule, which is not random. Other ships in categories 1 to 5, which do not have AIS, usually behave according to a random distribution.

In order to evaluate the proposed vessel counting system and provide its accuracy in capturing the total volume of maritime traffic, the overall vessel counting accuracy is calculated using the total vessel count from Table 5. The maximum counting accuracy achieved by the system is 98.23% while the minimum is 95.88%.

### 3.3. Runtime Analysis

**Figure 9:**
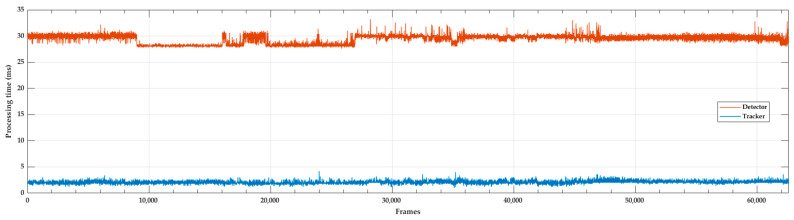
Tracker and detector processing time during the video sequence.

To obtain a general understanding of the processing speed of the proposed counting system (measured in milliseconds) on our laboratory computer (specifications provided in Table 1), processing speed data were collected during various stages of the system runtime. They include periods with lower numbers of vessels up to 6 vessels per frame. The total number of evaluated frames was 62,500, equivalent to 42 min of system runtime. The results are presented in Figure 9. The KF with HA multi-target tracker achieved an average processing speed of 2.07 ms per frame, with a minimum of 1 ms and a maximum of 12 ms. Meanwhile, the YOLOv4 detector achieved an average processing speed of 29.40 ms per frame, with a minimum of 27 ms and a maximum of 45 ms. Overall, counting system achieved average processing speed of 31.47 ms per frame, with a minimum of 29 ms and a maximum of 47 ms.

## 4. Discussion

While there are limited existing papers in the field of maritime vessel counting, the results and implications of this paper contribute to this niche area. The inspiration for our research comes largely from the ARGOS project [4,5], one of the first projects in the Mediterranean for video-based monitoring of maritime traffic, which used static, land-based cameras. The ARGOS system achieved a count accuracy over 80% using a conventional (non-NN) object classifier. However, it had problems tracking fast-moving vessels and was not assessed against the actual number of vessels passing through the monitored zone. The authors in [6] used an NN-based object detector and classifier for vessel counting in waterway scenes, but it was trained for a single general class for all vessels. In [7], on the other hand, the authors trained their system for five large classes of vessels, which do not match the maritime traffic profile of passenger ports.

This paper highlights the need for accurate and comprehensive vessel counting in passenger ports, specifically in Mediterranean ports such as Split. These ports are characterized by a large number of small vessels that are not monitored by conventional systems. Therefore, we propose an improved solution that effectively addresses these challenges, thus providing a more comprehensive and accurate vessel counting system.

The statistical analysis of vessel counts in Table 5 provides information on the range, central tendency, and variability between measurements of vessel counts within each category, highlighting several important results. It is also important to consider the data from Table 4, which does not summarize counts separately for vessels entering and leaving the port. The categories ‘Sailing Boat’, ‘Pleasure yacht’, ‘Medium Ferry’, ‘Large Ferry’, ‘High Speed Craft’, ‘Small Passenger ship’, and ‘Large Passenger Ship’ demonstrate high count accuracy. The counts for these categories closely match the GT, indicating accurate classification and counting. The high accuracy for these vessel categories can be attributed to their larger size, making them relatively easy to detect and classify by the detector. However, the ‘Sailing Boat’ category presents a different scenario. They often cross the counting zone farther away in the image, causing them to appear as medium-sized and sometimes even small objects in the image. This highlights the importance of having unique class features, such as sails, for reliable classification.

On the other hand, the category ‘Small Passenger Ship’ shows some discrepancy in counts (Table 4) while maintaining very high accuracy since classification, and therefore, counting errors seem to cancel each other out. In this particular case, the vessel ‘Small Passenger Ship’ was misclassified when leaving the port and counted for a certain category. Upon entering the port, another vessel was incorrectly classified and counted for the ‘Small Passenger Ship’ category. A high interclass similarity can explain this, as some vessel instances share visual features with several classes, leading to different classification results and, consequently, inaccuracies in the number of categories. This can also explain the results for ‘Motorboat’, which show a deviation from the GT and a counting accuracy of 83.06%. The facts observed here indicate that the counting result may contain errors that compensate for each other. For example, the counting error is zero if the number of FP equals to the number of FN for an observed category.

In addition, as mentioned earlier, the size of the objects in the image is important, especially for vessel categories, which are generally small. It is valid for the categories ‘Small craft’, ‘Small fishing boat’, and ‘Speed craft’. This factor becomes even more apparent when they are farther away in the image, causing their size to appear much smaller in the image. This leads to problems in detection or classification and, consequently, to lower counting accuracy. It should also be noted that the ‘Large Passenger ship’, the ‘Large Ferry’, and the ‘Small Fishing boat’ were the least common vessels in the test video. On the other hand, the ‘Trawler boat’ did not occur in the test video and was not counted by the counting system, which is also valuable information.

Possible effects of unbalanced training data could contribute to counting errors. Looking at Figure 2a, one might think that ‘Speed craft’ would achieve the highest counting accuracy because they are the most frequently represented category. However, based on the facts already mentioned, this is not the case. It should be noted that the ‘Sailing Boat’ category was the second most represented class in the training set and achieved a counting accuracy of 100%. Although the ‘Fishing trawler’ did not appear in the test video, it was the least represented class in the training set, and no vessel was misclassified.

One of the contributions is the tracking stability assessment, which complements the multi-target tracking algorithm. In Figure 7b, it can be seen that, due to the proposed improvement, the FP detection is eliminated and not tracked. However, the drawback of this improvement is that the TP track was not tracked in the first 30 frames. If we approximate the distance from the left or right side of the camera FoV (Figure 6) to the count zone, we can calculate the velocity that the vessel must reach to avoid the count zone. Since the camera stream has a speed of 25 FPS, the approximate speed the vessel must reach is 83 m/s. For the vessels that emerge on the left or right side of Figure 6 and then move toward the counting zone, we can assume that this improvement has no effect on the vessel count since only the fastest racing boats can reach these speeds.

However, it should be noted that if there is an occlusion between two ships, there is a possibility that the vessel that leaves the occlusion and then enters the counting zone will not be counted. Since the total number of vessels in the GT is 169 and the system’s maximum ship count is 167, this could indicate that the tracking stability assessment has removed a high percentage, if not all, of FP detections that are from unwanted objects (any detection that is not a vessel).

Finally, the average speed of the counting system is 31.78 FPS, with a peak performance of up to 34.48 FPS and a minimum of 21.28 FPS, which meets the requirements for our video stream provided in 25 FPS. To emphasize the accuracy of the proposed vessel counting system in capturing the total volume of maritime traffic, the overall average accuracy of vessel counting by the system is 97.76%. Comparing the manual count results from the AIS (Table 2) with the average total vessel count (Table 4), the proposed system recorded 386% more traffic than AIS during the same period. These results highlight the value of the SPSCD dataset for NN training and prove its potential for future scientific applications in maritime zones characterized by a similar traffic profile.

## 5. Conclusions

This paper proposes a ship counting system for monitoring and counting maritime traffic in Mediterranean ports, focusing on the Port of Split, Croatia. Given the diversity of maritime traffic in these areas, from small boats, sailboats, and speedboats to small passenger ships, ferries, and large cruise ships, conventional traffic monitoring systems often do not provide comprehensive data. The proposed system uses the YOLOv4 convolutional NN and Kalman multi-target tracker and achieves an average counting accuracy of 97.76% and an average processing speed of 31.78 FPS, highlighting its suitability for real-time applications and robustness.

However, the system can have counting errors due to misclassifications, which may potentially cancel each other out. Classifying vessels in such environments is challenging due to several factors, such as diverse vessel types, lack of standardized visual features, heavy occlusions, and variable weather and lighting conditions. In addition, system performance is limited in complete darkness. Sensor fusion should be considered to add a new mode, i.e., enable effective monitoring of night-time traffic, such as integrating an infrared camera. Addressing these issues is a potential area for future improvement.

Nevertheless, the system recorded 386% more maritime traffic data than the conventional AIS system. This highlights the significant potential of the proposed system to facilitate the acquisition of detailed and accurate maritime traffic data for scientific research in similar maritime zones.

## Figures and Tables

**Table 1:** Computer specifications.

Processor	Intel Core i7-9700K @ 3.6 GHz
Memory	32 GB RAM, DDR4 3200
Storage	500 GB NVMe M2 SSD
GPU	NVIDIA GeForce RTX 2080, 8 GB RAM
OS	Windows 10

**Table 2:** Results obtained using multiple metrics.

COCO Metrics									PASCAL
AP	AP@0.50	AP@0.75	AR_1_	AR_10_	AR_100_	AP_S_	AP_M_	AP_L_	mAP
0.6576	0.9246	0.8170	0.6481	0.7066	0.7066	0.3280	0.6366	0.7415	0.9277

**Table 3:** AIS manual vessel counting.

AIS Vessel Category	Entering the Port	Leaving the Port	Total Count
Passenger (A)	6	10	16
Hi-Speed Craft	4	7	11
Pleasure Craft (A)	1	1	2
STBD Marker ()	1	0	1
Unspecified (A)	1	1	2
Unspecified ()	1	1	2

**Table 4:** Manual vessel counts and the best result from the counting system.

Vessel Category	Manual Counting			Best System Results		
	Entering	Leaving	Total	Entering	Leaving	Total
Small craft	5	6	11	3	6	9
Small fishing boat	0	1	1	1	2	3
Sailing boat	6	10	16	6	10	16
Speed craft	27	50	79	25	48	73
Motorboat	4	7	11	5	9	14
Pleasure yacht	3	6	9	3	7	10
Medium ferry	4	4	8	4	4	8
Large ferry	0	2	2	0	2	2
High speed craft	4	7	11	4	7	11
Small passenger ship	5	15	20	4	16	20
Fishing trawler	0	0	0	0	0	0
Large passenger ship	1	0	1	1	0	1
Total vessel count	59	108	169	56	111	167

**Table 5:** Statistical characteristics of vessel count for each vessel category.

Vessel Category	GT	Min	Max	Average	Median	STD	Counting Accuracy
Small craft	11	9	10	9.03	9	0.18257	82.12%
Small fishing boat	1	3	3	3.00	3	0	−100.00%
Sailing boat	16	16	16	16.00	16	0	100.00%
Speed craft	78	72	73	72.57	73	0.50400	93.03%
Motorboat	12	13	15	14.03	14	0.41384	83.06%
Pleasure yacht	9	8	10	8.97	9	0.41384	99.63%
Medium ferry	8	8	8	8.00	8	0	100.00%
Large ferry	2	2	2	2.00	2	0	100.00%
High speed craft	11	10	11	10.90	11	0.30512	99.09%
Small pass. ship	20	19	20	19.80	20	0.40683	99.00%
Fishing trawler	0	0	0	0.00	0	0	-
Large pass. ship	1	1	1	1.00	1	0	100.00%
**Total vessel count**	169	163	167	165.30	165	0.95231	97.76%

**Figure A1:**
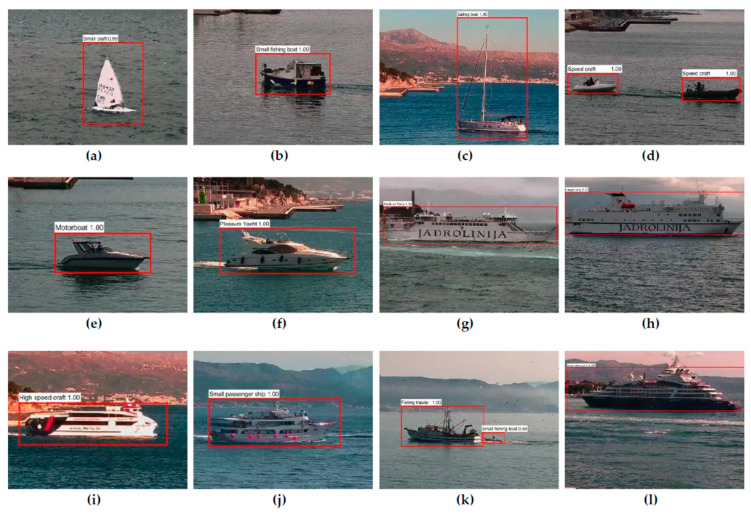
Vessel images from each class, depicted as follows: (**a**) ‘Small craft’ (**b**) ‘Small fishing boat’; (**c**) ‘Sailing boat’; (**d**) are two ‘Speed craft’ vessels; (**e**) ‘Motorboat’; (**f**) ‘Pleasure Yacht’; (**g**) ‘Medium ferry’; (**h**) ‘Large ferry’; (**i**) ‘High-speed craft’; (**j**) ‘Small Passenger ship’; (**k**) presents a ‘Fishing trawler’ on the left and a ‘Small fishing boat’ on the right; and finally (**l**) a ‘Large Passenger ship’.

**Figure A2:**
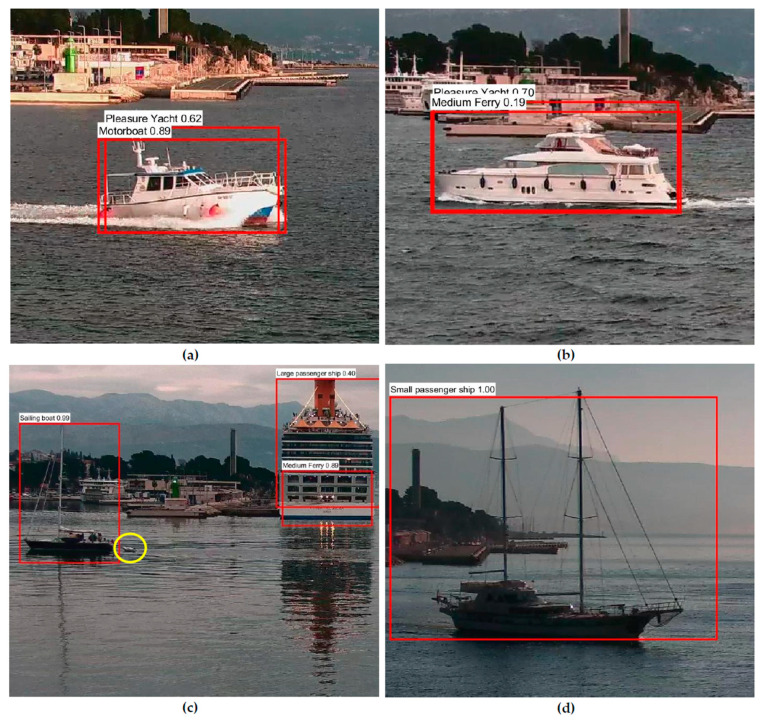
Example images demonstrating various prediction errors: (**a**) shows the ‘Motorboat’ class vessel with additional incorrect ‘Pleasure Yacht’ prediction; (**b**) shows the ‘Pleasure Yacht’ class vessel with additional incorrect ‘Motorboat’ prediction; (**c**) shows the ‘Sailing boat’ class vessel on the left side of the image, while on the right there is a ‘Large Passenger ship’ with additional incorrect ‘Medium ferry’ prediction. Furthermore, an undetected ‘Small craft’ vessel is marked with a yellow circle; (**d**) represents a larger ‘Sailing boat’ class vessel incorrectly labeled as ‘Small Passenger ship’.

## Data Availability

The Split Port Ship Classification Dataset (SPSCD) and video sequence used for the counting system evaluation can be downloaded from: https://labs.pfst.hr/maritime-dataset/ (accessed on 1 February 2023) and used for scientific purposes only.

## Appendix A

This appendix serves as a supplement to Section 2.2. The images are extracted from the validation set after evaluation, and thus, bounding boxes are drawn around the vessels. Additionally, the images are enlarged for better viewing.
